# Enhanced grading methods for lumbar paraspinal fat infiltration and its prognostic value in predicting lumbar disc herniation

**DOI:** 10.1186/s13018-023-04247-w

**Published:** 2023-10-04

**Authors:** Gang Wen, Wanmei Hou, Guangwei Xu

**Affiliations:** 1 Department of Emergency, Nanfang Hospital, Southern Medical University, Guangzhou, 510515 Guangdong China; 2 Southern Medical University, Guangzhou, 510515 Guangdong China

**Keywords:** Paraspinal muscles, Intervertebral disc degeneration, Magnetic resonance imaging, Low back pain, Adipose tissue

## Abstract

**Background:**

The simplified 3-grade system for measuring fat infiltration in the paraspinal muscles is widely utilized. In comparing our proposed 4-grade system to the existing 3-grade system, we evaluated its impact on results and particularly its ability to predict disc herniation, ultimately highlighting deficiencies in the latter. The objective of this investigation was to validate the efficacy of our newly proposed semi-quantitative simplified 4-grade system for assessing fat infiltration, as compared to the existing literature-based simplified 3-grade system, in terms of their predictive value for lumbar disc herniation.

**Methods:**

Infiltration of the right and left lumbar multifidus and erector spinae muscles were assessed using a semi-quantitative 3- and 4-grade fat infiltration system on axial magnetic resonance imaging sections at the L3-S1 level in all subjects, with comparison of results between groups. The correlation between these grading systems and lumbar disc herniation was investigated.

**Results:**

The simplified 3-degree system for measuring fat infiltration was not effective in predicting lumbar disc herniation (*p* > 0.05), while the 4-degree system proved to be useful in predicting it (*p* < 0.05). In both grading systems, females were found to have a higher risk of lumbar disc herniation than males (*p* < 0.05), and the risk increased with age and body mass index (BMI) (*p* < 0.001).

**Conclusions:**

It was observed that using the 4-grade fat infiltration system to determine the level of fat infiltration in the paraspinal muscles is more effective in predicting lumbar disc herniation compared to the 3-grade system. The 4-grade fat infiltration grading system proves to be an efficient semi-quantitative method that can replace the simplified 3-grade system.

## Background

Lumbar disc herniation is a prevalent condition affecting the lumbar spine, which can result in back pain, muscle spasms, and limited mobility [1–3]. The paraspinal muscles play a crucial role in both functional and structural stabilization of the lumbar spine. The superficial layer is primarily composed of large back muscles responsible for spinal and limb movements, while the segmental layer consists of deep muscles that control intersegmental movement [4]. Zhao et al. [5] retrospectively analysed the morphological characteristics of paraspinal muscles in young patients with unilateral neurological symptoms of lumbar disc herniation, concluded that greater muscle atrophy on the normal side of the multifidus increased the incidence of low back pain in young patients with unilateral lumbar disc herniation. Kuligowski [6] considered that the different types of lumbar disc herniation do not affect the prevalence of lumbar segmental instability in young individuals. However, many literatures have reported that polyfidus atrophy is associated with lumbar disc herniation and lumbar postoperative pain [7–9]. Non-invasive methods, such as Computed Tomography (CT), Magnetic Resonance Imaging (MRI), ultrasound, Magnetic Resonance Spectroscopy, chemical shift MRI and multi-core MRI can be utilized to measure fat infiltration of paraspinal muscles [4]. Fat infiltration can be evaluated through qualitative, semi-quantitative, and quantitative methods [4]. MRI offers high-resolution, high-contrast imaging of soft tissue without exposing patients to radiation. Moreover, its reliability has been reported to surpass that of CT scanning [10–12]. Goutallier et al. [13] initially demonstrated a qualitative assessment of the rotator cuff muscle system by visually grading fatty infiltration on CT scans using a five-point scale. With the advancement of MRI technology, Fuchs et al. [14] conducted a comparative analysis of rotator cuff fatty infiltration using both CT and MRI evaluations, resulting in the simplification of the original five-grade rating system. Slabough and Solgaard Sorenson et al. [15, 16] evaluated the extent of fat infiltration in adults using standard visual assessment and categorized it as normal, mild or severe based on the involvement of one or more lumbar segments. Parkkola et al. [17] visually classified the degree of fat infiltration into four grades: normal, mild, significant and severe. Some scholars quantify it by percentage ratio and simplify the 3-level system evaluation. Therefore, many authors have begun to quantitatively measure the fat infiltration of the paraspinal muscles [17–28].

Based on the hypothesis that this may create weaknesses and may be insufficient to obtain detailed results, the aim of this study was to evaluate the 4-grade fat infiltration (< 10%, 10–30%, 30–50%, > 50%) measurement method and its sensitivity as a new alternative method.

## Method

Patients who were admitted to an orthopaedics and traumatology department with complaints of low back pain and diagnosed with lumbar disc herniation between January 2022 and December 2022 were invited to participate in the study. Total of 40 patients were recruited and included in the patient group, while a control group consisting of 40 healthy individuals was randomly selected from volunteers who responded to social media and announcements. The control subjects had not experienced back pain within the past year and exhibited no signs of back problems during examination or radiology. The group of patients included those under 70 years old who had been experiencing low back pain for the past three months and were diagnosed with disc herniation. Exclusion criteria for the study consisted of a diagnosis of root compression (radiculopathy) on MRI, any rheumatological or infectious disease, spinal or hip deformities, a history of lumbar surgery, or acute pain in another part of the body. EMG was requested for patients suspected of having root compression, and they were included in the study only after it was confirmed that there was no such compression. Demographic and disease-related information was collected through face-to-face interviews using a demographic information form. Hemogram, erythrocyte sedimentation rate, complete urinalysis, ASO, CRP, RF, salmonella and brucella tests were conducted as needed to aid in differential diagnosis. The same orthopaedic and traumatology specialist evaluated all 80 subjects, while an experienced consultant radiologist analysed the lumbar spine MRI images without knowledge of their clinical history. All MRI scans were conducted by the same technician, and participants provided informed consent before being included in the study. Unit Ethics Committee 22 (SDU-2022-12107) approved the study.

A 3.0 Tesla MRI device was used for image acquisition. The patient was placed in a supine position with a knee pillow and a spine coil. Based on laser localization, the measurement level between L3-S1 (L3-4/L4-5/L5-S1) was directed towards the lumbar spine without any tilting to either side, with the centre of the disc being the measurement level. All the measurements were taken with a routine protocol. For the measurements, turbo spin-echo T1 and T2-weighted sagittal and turbo spin-echo T2 axial 4 mm sections parallel to the disc spaces were taken. Evaluations were made on T2 axial sections. The fat contents of the erector spinae (miliocostalis + mlongissimus) and multifidus muscles at L3-S1 level were evaluated at all 3 levels, on the right and left.

The degree of fat infiltration in the muscles was assessed using two semi-quantitative grading systems: a simplified 3-grade system and a 4-grade system. Under the simplified 3-grade system, grade 1 indicated normal muscle condition with up to 10% fat infiltration of the muscle surface cross-sectional area, grade 2 indicated moderate condition with fat infiltration ranging from 10 to 50%, and grade 3 indicated severe condition with over 50% fat infiltration [3]. The simplified 4-grade system was assessed as follows: Grade 1 indicates normal muscle condition with up to 10% fat infiltration of the muscle surface cross-sectional area; Grade 2 denotes mild degree with fat infiltration ranging from 10 to 30%; Grade 3 represents moderate level with fat infiltration between 30 and 50%; and finally, Grade 4 signifies severe condition where more than half of the muscle is infiltrated by fat.

For the purpose of reliability evaluation, a total of 10 patients were randomly selected and re-evaluated by the same radiologist (K.T) after one month. The resulting kappa values for intra-observer agreement were 0.934 and 0.921 for the simplified three-grade system and four-grade system, respectively.

The data were analysed using IBM SPSS 21, and binary logistic regression analysis was employed to identify the risk factors associated with lumbar disc herniation. The results of the analysis were presented as odds ratios (95% confidence intervals). In the univariate analysis, each variable was entered into the model separately to assess its individual effect. In the multivariate analysis, all variables were simultaneously entered into the model and their combined effects were examined. Statistical significance was set at *p* < 0.05. Based on a 95% confidence level (1−*α*), 95% test power (1−*β*), and degeneration values of 0.32 and 0.80, a minimum sample size of 50 was determined for inclusion in the study. To account for potential participant attrition, a total of 60 subjects were enrolled to complete the study.

## Results

### 3-Grade classification system

The demographic and physical characteristics of the patients, as presented in Table 1, were analysed to examine fat infiltration of the paraspinal muscles in both control and patient groups using a simplified 3-grade classification (Table 2). Results from univariate (OR = 2.033; *p* = 0.03) and multivariate (OR = 4.125; *p* = 0.002) analyses indicated that females had a higher risk for lumbar disc herniation compared to males, the risk of lumbar disc herniation was observed to increase with age (OR = 1.111–1.354; *p* < 0.001). However, in both univariate and multivariate analyses, the right-left multifidus muscles and right-left merector spinae muscles fat infiltration classes were not identified as independent risk factors (*p* > 0.05). Furthermore, no significant association was found between fat infiltration and lumbar disc herniation in this grading system.

**Table 1:** Comparison of the demographic characteristics of the groups

	Control group		Patient group		Total		*p*
	*x̅* ± σ	Median (min–max)	*x̅* ± σ	Median (min–max)	*x̅* ± σ	Median (min–max)	
Age	36.4 ± 7.29	35 (27–58)^a^	44.5 ± 5.17	42.6 (23–67)^b^	36.4 ± 8.1	44 (23–67)	** < 0.001**^1^
BMI	24.81 ± 3.38	25.42 (18.23–33.70)^ab^	22.07 ± 4.73	25.01 (20.34–37.2)^b^	27.5 ± 3.4	27.8 (18.23–37.2)	** < 0.001**^1^
Duration of							
back pain	–	–	49.1 ± 54.02	37 (4–230)			
VAS at rest	–	–	2.67 ± 1.18	2 (1–7)			
VAS activity	–	–	4.53 ± 1.64	4 (1–9)			
Gender	*n* (%)		*n* (%)				
*Male*	23 (57.5)		18 (45)		41 (51.3)		0.289^2^
*Female*	17 (42.5)		22 (55)		39 (48.7)		

^1^Kruskal Wallis, ^2^Mann Whitney U, ^2^Pearson Chi Square, a-b-ab: There is a difference between groups with different superscript letters for each measurement value

**Table 2:** Determination of risk factors affecting lumbar disc herniation by logistic regression with simplified 3- degree fat infiltration system

	Univariate		Multivariate	
	OR (95% CI)	*p*	OR (95% CI)	*p*
Gender (female)	2.033 (1.214–3.823)	**0.030**	4.125 (1.765–9.819)	**0.002**
Age	1.611 (1.111–1.354)	** < 0.001**	1.163 (1.232–1.131)	** < 0.001**
BMI	1.165 (1.116–1.165)	** < 0.001**	1.177 (0.945–1.522)	0.216
Right musculus multifidus*	1.781 (0.914–2.907)	0.059	2.626 (0.615–9.895)	0.113
Left musculus multifidus*	1.431 (0.667–2.432)	0.159	1.453 (0.536–5.94)	0.402
Right musculus erector spina*	1.644 (0.664–2.425)	0.509	0.590 (0.143–2.762)	0.460
Left musculus erector spina*	1.763 (0.623–2.842)	0.665	0.871 (0.165–3.843)	0.750

*Ratings of < 10%; 10–50%; > 50% were used in the evaluation

### 4-Grade classification system

After examining fat infiltration according to the 4-grade classification of both control and patient groups (Table 3), univariate analysis revealed that females had a 2.10-fold higher risk of lumbar disc herniation compared to males when males were taken as the reference in the gender variable (*p* = 0.020). When using the right multifidus muscle value as a reference for < 10%, individuals with a fat infiltration rate between 10 and 30% had a 3.75-fold higher risk of lumbar disc herniation, while those with a 30–50% risk did not differ significantly from those with a 10% risk. The left multifidus and right erector spinae muscles were not found to be significant risk factors for lumbar disc herniation (*p* values of 0.502 and 0.105, respectively). When using < 10% as a reference for left erector spinae fat infiltration, individuals with 10–30% had a 2.595-fold higher risk of lumbar disc herniation (*p* = 0.024), while those with 30–50% had a 5.13-fold higher risk (*p* = 0.005).

**Table 3:** Determination of risk factors affecting lumbar disc herniation by logistic regression with simplified 4-grade fat infiltration system

	Univariate		Multivariate	
	OR (95% CI)	*p*	OR (95% CI)	*p*
Gender (female)	2.213 (1.324–3.763)	**0.020**	3.721 (1.475–8.874)	**0.005**
Age	1.811 (1.121–1.324)	**0.000**	1.315 (1.321–1.344)	**0.000**
BMI	1.137 (1.826–1.135)	**0.000**	1.726 (0.448–1.868)	0.275
Right musculus multifidus*	**Referance: < 10%**		1.450 (0.413–4.659)	0.323
10–30%	3.754 (1.501–8.120)	**0.002**		
30–50%	2.630 (0.755–9.656)	0.723		
Left musculus multifidus*	1.321 (0.731–2.436)	0.502	0.982 (0.302–3.655)	0.898
Right musculus erector spina*	1.968 (0.741–2.582)	0.105	0.770 (0.355–1.92)	0.095
Left musculus erector spina*	**Reference: < 10%**		**Reference: < 10%**	
10–30%	2.595 (1.320–5.366)	**0.024**	5.654 (1.311–23.543)	**0.037**
30–50%	5.135 (1.625–15.565)	**0.005**	14.238 (0.863–136.142)	0.077

*Ratings of < 10%; 10–30%; 30–50%; > 50% were used in the evaluation

**Fig. 1:**
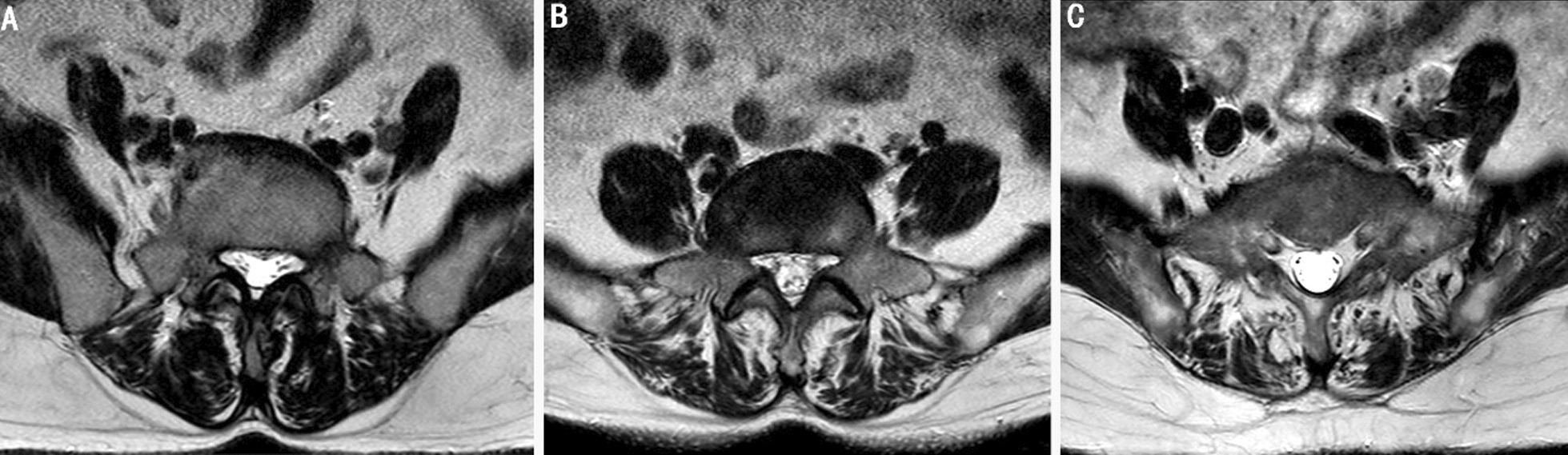
Muscular location and fat infiltration degrees in cross section with T2 axial Magnetic Resonance Imaging (evaluation with simplified 3-grade system) **A** Grade 1 =  < 10% fat infiltration, **B** Grade 2 = 10–50% fat infiltration, **C** Grade 3 =  > 50% fat infiltration

**Fig. 2:**
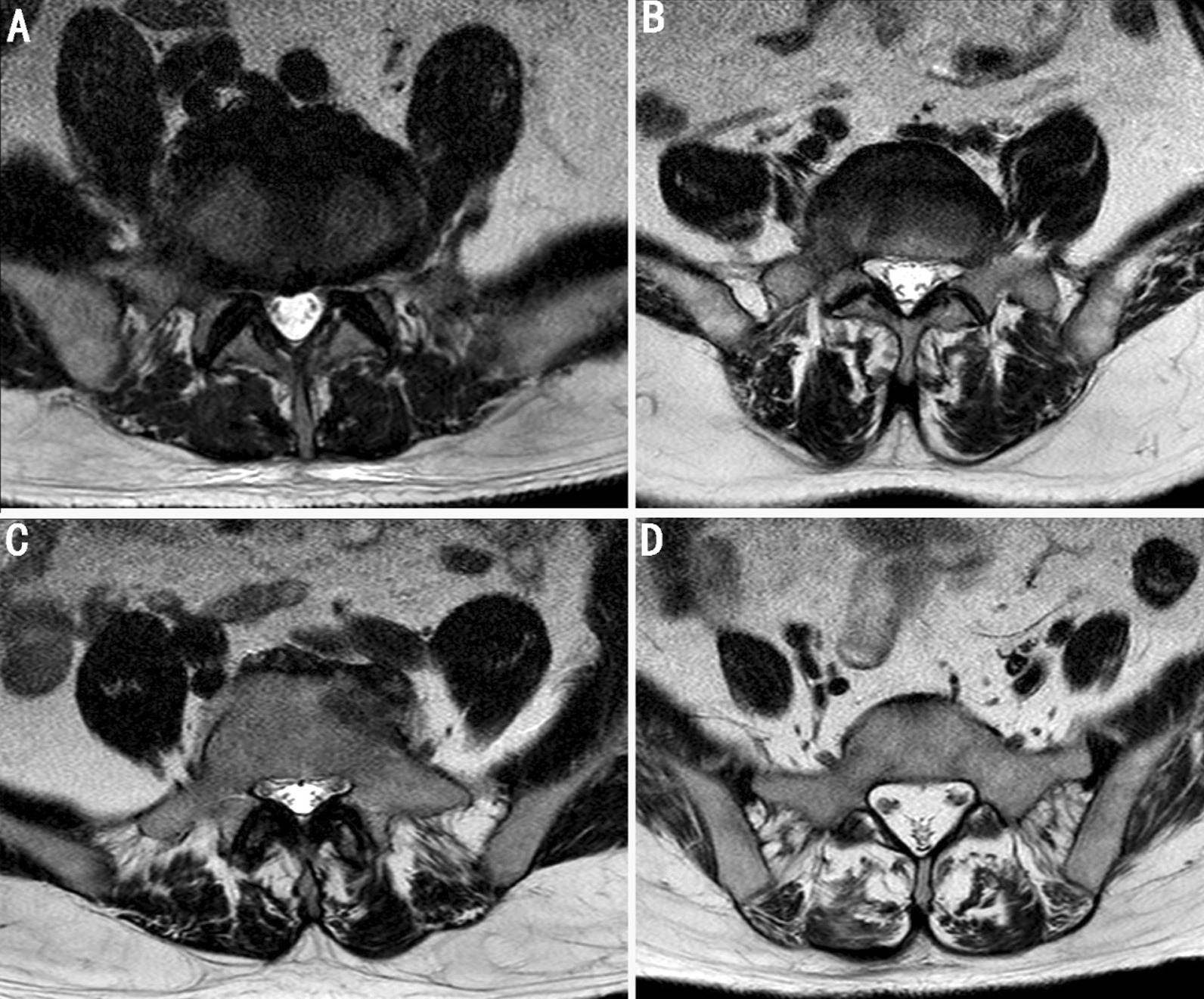
Muscular location and fat infiltration grades on T2 axial MRI slice (evaluation with 4 grade system) **A** Grade 1 =  < 10% fat infiltration, **B** Grade 2 = 10–30% fat infiltration, **C** Grade 3 = 30–50% fat infiltration, **D** Grade 4 =  > 50% fat infiltration

Although the images in Figs. 1B and 2B are identical, the measurement results differ between the 3-grade system (10–50%) and the 4-grade system (10–30%). Multivariate analysis revealed that females have a 3.72-fold higher risk than males, while age was found to increase lumbar disc herniation risk by 1.31-fold (*p* = 0.000). With reference to less than 10% of the left merector spina, individuals with 10–30% fat infiltration had a 5.654-fold higher risk of lumbar disc herniation, which could increase up to 23.54-fold (*p* = 0.037). The simplified four-grade system demonstrated a strong correlation between fat infiltration and lumbar disc herniation (Table 4).

**Table 4:** The predictive strength of simplified 3 and 4 grade fat infiltration systems in lumbar herniation outcome

Effect (strength) in predicting hernia	
Simplified 3 degree fat infiltration system	
Grade 1: 10% < fat infiltration (normal)	nA
Grade2: 10–50% fat infiltration (medium)	nA
Grade 3: 50% > fat infiltration (severe)	nA

Simplified 4 degree fat infiltration system	
Grade 1: 10% < fat infiltration (normal)	nA
Grade 2: 10–30% fat infiltration (light)	5.18 (1.31–22.53) times
Grade 3: 30–50% fat infiltration (medium)	5.22 (1.54–17.59) times
Grade 4: 50% fat infiltration ((severe)	nA

nA—not applicable

## Discussion

The primary discovery of this investigation indicates that the simplified 4-grade system for fat infiltration is more efficacious in predicting lumbar disc herniation when compared to the simplified 3-grade system. Diverse viewpoints have been expressed concerning the paraspinal muscle's fat infiltration, which may be attributed to the limitations of various measurements. Therefore, more precise methods of measurement are required. The objective of this study was to establish a simplified 4-grade measurement system and determine its sensitivity. Currently, there is no existing definition for the simplified 4-grade system in literature.

Various techniques are employed for assessing fat infiltration, and the simplified 3-grade system utilizing the qualitative Goutallier method has demonstrated good intra- and inter-observer reliability in semi-quantitative evaluations [21, 30]. Kjaer et al. [18] found that while both intraobserver and interobserver reliability were satisfactory, visual assessment of fat infiltration did not produce satisfactory results for adolescents. Therefore, relying solely on visual examination to determine the degree of fat infiltration in muscles is insufficient [4]. Although fat infiltration in the 10–50% range of the simplified 3-grade system does not provide insight into the risk of lumbar disc herniation, a significant correlation between fat infiltration and this risk was observed within the 10–30% and 30–50% ranges of the more detailed 4-grade system that corresponds to the aforementioned range. Notably, individuals with fat infiltration levels between 10–30% exhibit up to a staggering 21.5-fold increase in their likelihood of developing lumbar disc herniation. It can be argued that the observed outcome is attributed to the wide range of 10–50% in the three-grade system, encompassing extreme values (15–45%). Therefore, it is crucial to subdivide this band into 10–30% and 30–50% sub-bands for accurate assessment of lumbar disc herniation risk. Fat infiltration appears to manifest as a late-stage muscle degeneration phenomenon, with lumbar multifidus muscle fat infiltration increasing with age among adults regardless of body composition [18]. Kidde et al. [31] proposed that the infiltration of fat into muscles may have a greater impact on mobility function than muscle weakness. Previous research has indicated a correlation between the infiltration of fat and herniation of lumbar discs [32–34]. In obese individuals, body fat tends to accumulate naturally in the muscles along the back musculature, but not typically at the level of the last two lumbar vertebrae where spinal problems are often prevalent. However, when there is a significant presence of fat infiltration in these specific problem areas, it suggests that back pain may be initiating muscle changes [4].

The morphological features of muscle degeneration include a decrease in muscle size (atrophy) and an increase in fat accumulation [17, 35]. Similar to the findings of the present investigation, numerous studies have demonstrated that individuals suffering from low back pain exhibit greater levels of fat infiltration compared to healthy asymptomatic individuals [18, 19, 30]. In the literature, there have been varying reports on the association between fat infiltration and herniated discs. While some studies suggest a correlation solely between multifidus muscle and fat infiltration, others have also observed that there is a correlation between the accumulation of fat in both the multifidus and erector spinae muscles [18, 19, 35]. Several studies have demonstrated no correlation between fat infiltration of the multifidus and erector spinae muscles. Longitudinal review studies have yielded inconclusive findings regarding the relationship between paraspinal muscle morphology and chronic low back pain. Specifically, conflicting results have been reported for fat infiltration of the multifidus muscle, while evidence pertaining to the erector spinae muscle is limited [29, 33, 36–38].

Several studies have indicated that the accumulation of fat in the multifidus muscle is linked to gender and age [19, 20]. Parkkola et al. [17] found that as people age, they are more likely to experience lumbar disc herniation and degeneration of the muscles surrounding the spine. In line with previous studies in both grading systems, the risk of lumbar disc herniation increases with age. When looking at gender differences, it has been found that females are twice as likely to experience lumbar disc herniation compared to males [39]. The current study also shows that females have a 2.1 times higher risk of lumbar disc herniation than males in both grading systems.

There are conflicting findings on the association between body mass index and fat infiltration. While some studies suggest a link between the two [24, 40]. The current study found that as body mass index increased in both grading systems, so did the risk of lumbar disc herniation. However, it should be noted that the number of cases with fat infiltration over 50% was limited, which is a limitation of this study.

## Conclusion

The findings of this study indicate that although the simplified 3-grade fat infiltration system is not a reliable predictor of lumbar disc herniation, a significant correlation was observed with the simplified 4-grade fat infiltration system. To effectively predict the risk of lumbar disc herniation, it is important to use a semi-quantitative simplified 4-grade fat infiltration system instead of a 3-degree one for measuring muscle fat infiltration.

## Data Availability

Data are available for reviewing upon request.
